# Supplementary material for: Novel Dihydrocoumarins Induced by Radiolysis as Potent Tyrosinase Inhibitors
Source: Molecules. 2024 Jan 10;29(2):341. doi: 10.3390/molecules29020341 (PMC10820468; doi:10.3390/molecules29020341)
Supplement: Supplementary file 1 [file molecules-29-00341-s001.zip › molecules-2818466-supplementary.pdf]

Supplementary Materials

Novel Dihydrocoumarins Induced by Radiolysis as Potent Tyrosinase Inhibitors

Gyeong Han Jeong [1,2], Manisha Yadav [3], Seung Sik Lee [1,4], Byung Yeoup Chung [1], Jae-Hyeon Cho [5], In-Chul Lee [6], Hyoung-Woo Bai [1,2,4,*], and Tae Hoon Kim [3,*]

[1] Research division for Biotechnology, Advanced Radiation Technology Institute (ARTI), Korea Atomic Energy Research Institute (KAERI), Jeongeup 56212, Republic of Korea; jkh4598@hanmail.net (G.H.J.); sslee@kaeri.re.kr (S.S.L.); bychung@kaeri.re.kr (B.Y.C.).

[2] Center for companion animal new drug development, Korea Institute of Toxicology (KIT), Jeongeup 56212, Republic of Korea

[3] Department of Food Science and Biotechnology, Daegu University, Gyeongsan 38453, Republic of Korea; manishayv41@gmail.com (M.Y.)

[4] Radiation Biotechnology and Applied Radioisotope Science, University of Science and Technology (UST), Daejeon 34113, Republic of Korea

[5] Institute of Animal Medicine, College of Veterinary Medicine, Gyeongsang National University, Jinju 52828, Republic of Korea; jaehcho@gun.ac.kr (J.H.C.)

[6] Department of Cosmetic Science and Technology, Seowon University, Cheongju 28674, Republic of Korea lic9418@seowon.ac.kr (I.C.L.)

* Correspondence: hbai@kaeri.re.kr (H.W.B.); skyey7@daege.ac.kr (T.H.K.)

Contents

Figure S1. ^{1}H NMR spectrum of compound **1** in CD_3OD.

Figure S2. ^{13}C NMR spectrum of compound **1** in CD_3OD.

Figure S3. HSQC spectrum of compound **1** in CD_3OD.

Figure S4. HMBC spectrum of compound **1** in CD_3OD.

Figure S5. NOESY spectrum of compound **1** in CD_3OD.

Figure S6. HRESIMS spectrum of compound **1**.

Figure S7. ^{1}H NMR spectrum of compound **2** in CD_3OD.

Figure S8. ^{13}C NMR spectrum of compound **2** in CD_3OD.

Figure S9. HSQC spectrum of compound **2** in CD_3OD.

Figure S10. HMBC spectrum of compound **2** in CD_3OD.

Figure S11. NOESY spectrum of compound **2** in CD_3OD.

Figure S12. HRESIMS spectrum of compound **2**.

Figure S13. ^{1}H NMR spectrum of compound **3** in CD_3OD.

Figure S14. ^{13}C NMR spectrum of compound **3** in CD_3OD.

Figure S15. HSQC spectrum of compound **3** in CD_3OD.

Figure S16. HMBC spectrum of compound **3** in CD_3OD.

Figure S17. NOESY spectrum of compound **3** in CD_3OD.

Figure S18. HRESIMS spectrum of compound **3**.

Figure S19. ^{1}H NMR spectrum of compound **4** in acetone-d_6+D_2O.

Figure S20. ^{13}C NMR spectrum of compound **4** in acetone-d_6+D_2O.

Figure S21. HSQC spectrum of compound **4** in acetone-d_6+D_2O.

Figure S22. HMBC spectrum of compound **4** in acetone-d_6+D_2O.

Figure S23. NOESY spectrum of compound **4** in acetone-d_6+D_2O.

Figure S24. HRESIMS spectrum of compound **4**.

Figure S25. HPLC chromatograms of gamma-ray treated reactant and newly generated compounds **1**–**4**.

Figure S26. Effects of isolated compounds on the cell viability in B16F10 cells. Cell viability detection by MTT assay in B16F10 cells after treatment with extracts for 24 h.

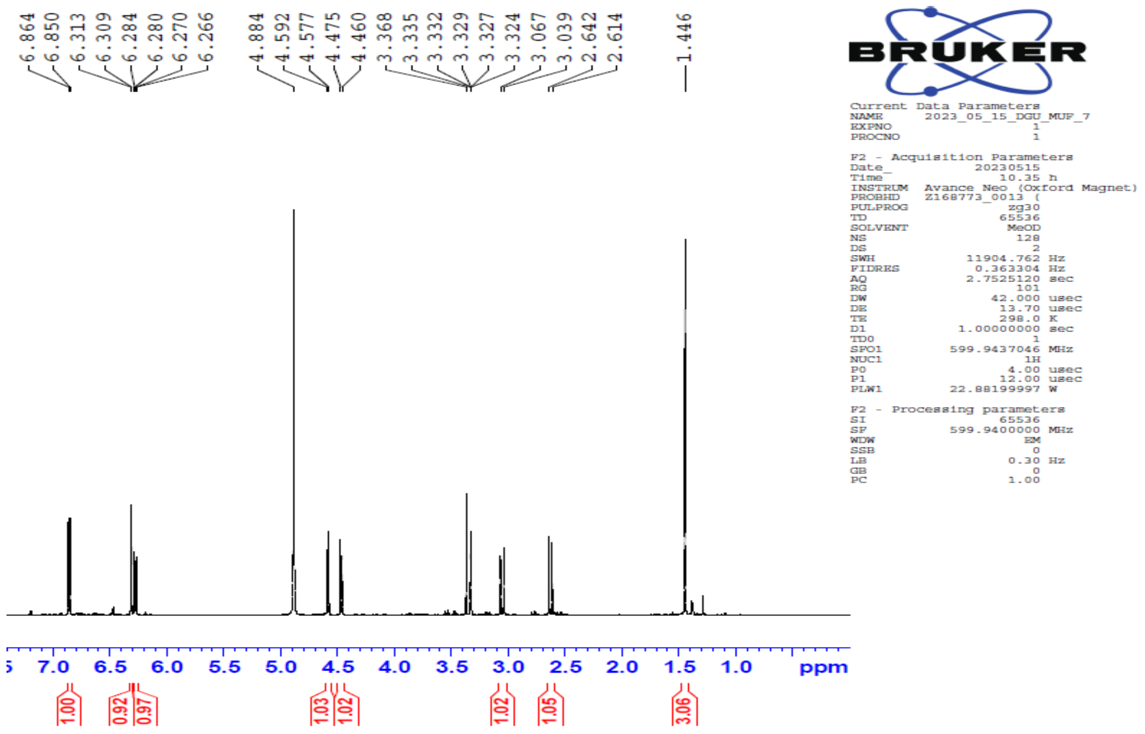

Figure S1. ^{1}H NMR spectrum of compound **1** in CD_3OD.

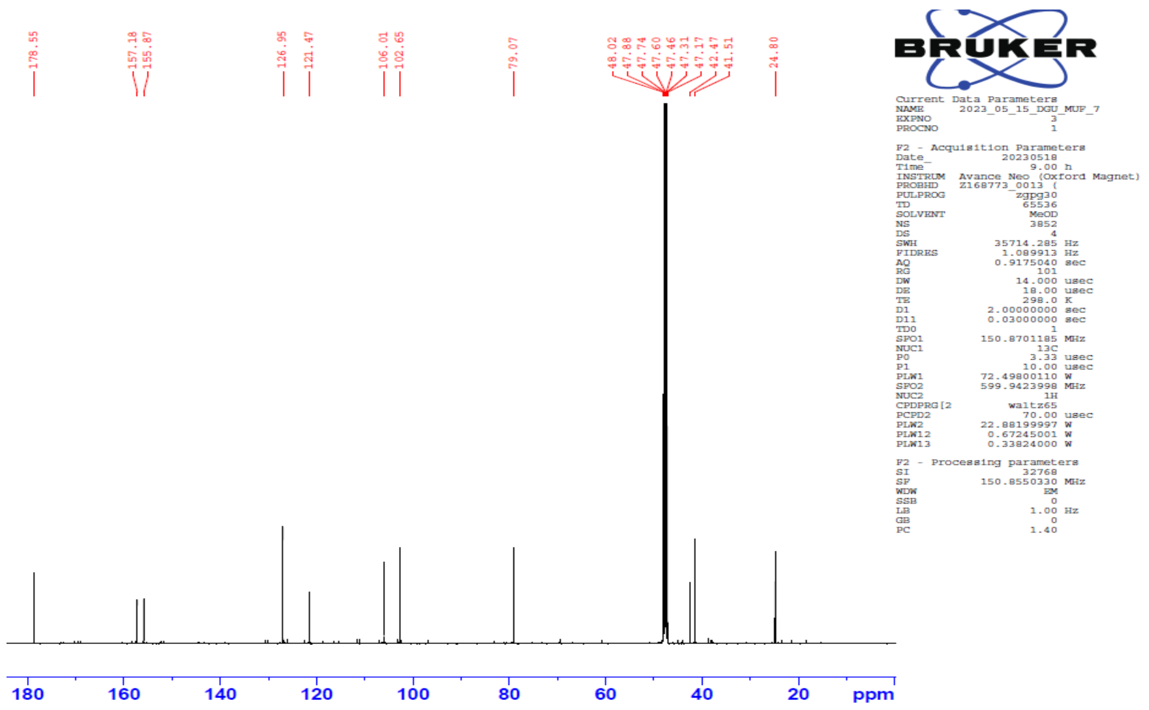

Figure S2. ^{13}C NMR spectrum of compound **1** in CD_3OD.

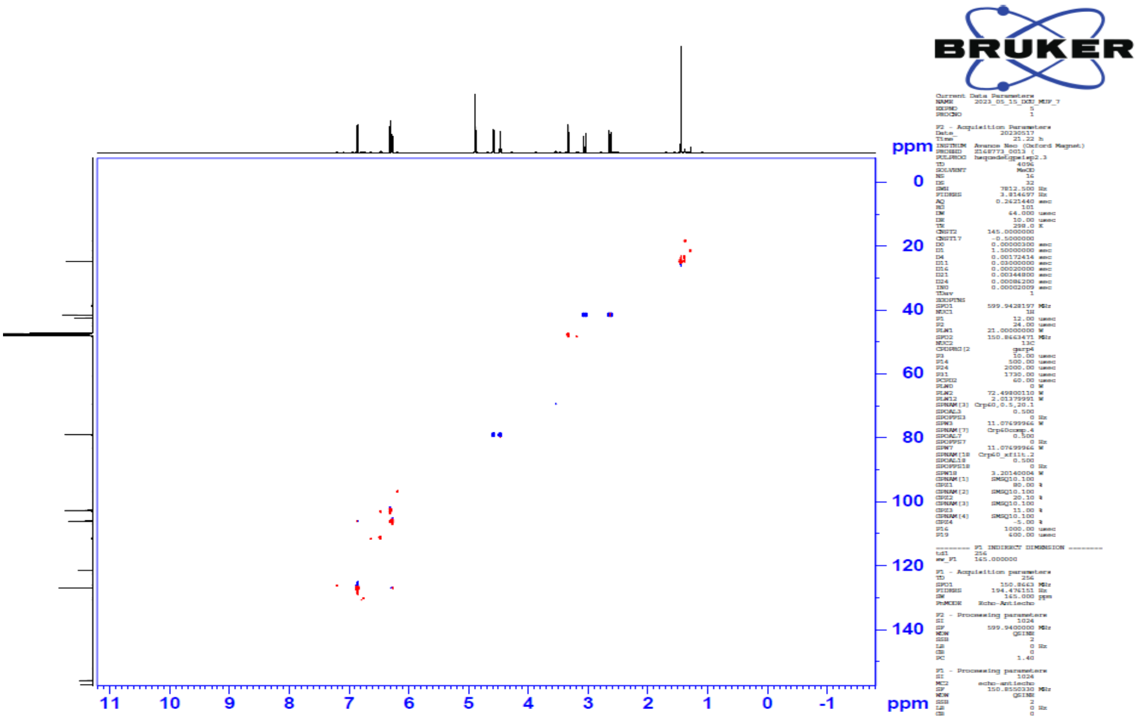

Figure S3. HSQC spectrum of compound **1** in CD_3OD.

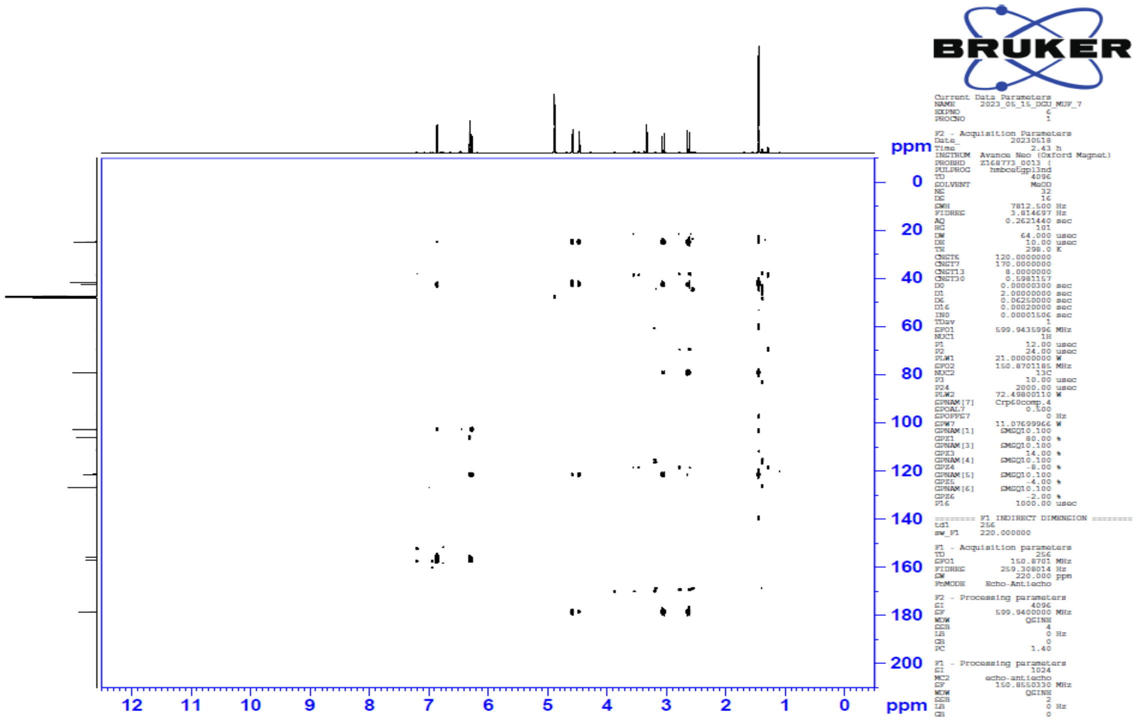

Figure S4. HMBC spectrum of compound **1** in CD_3OD.

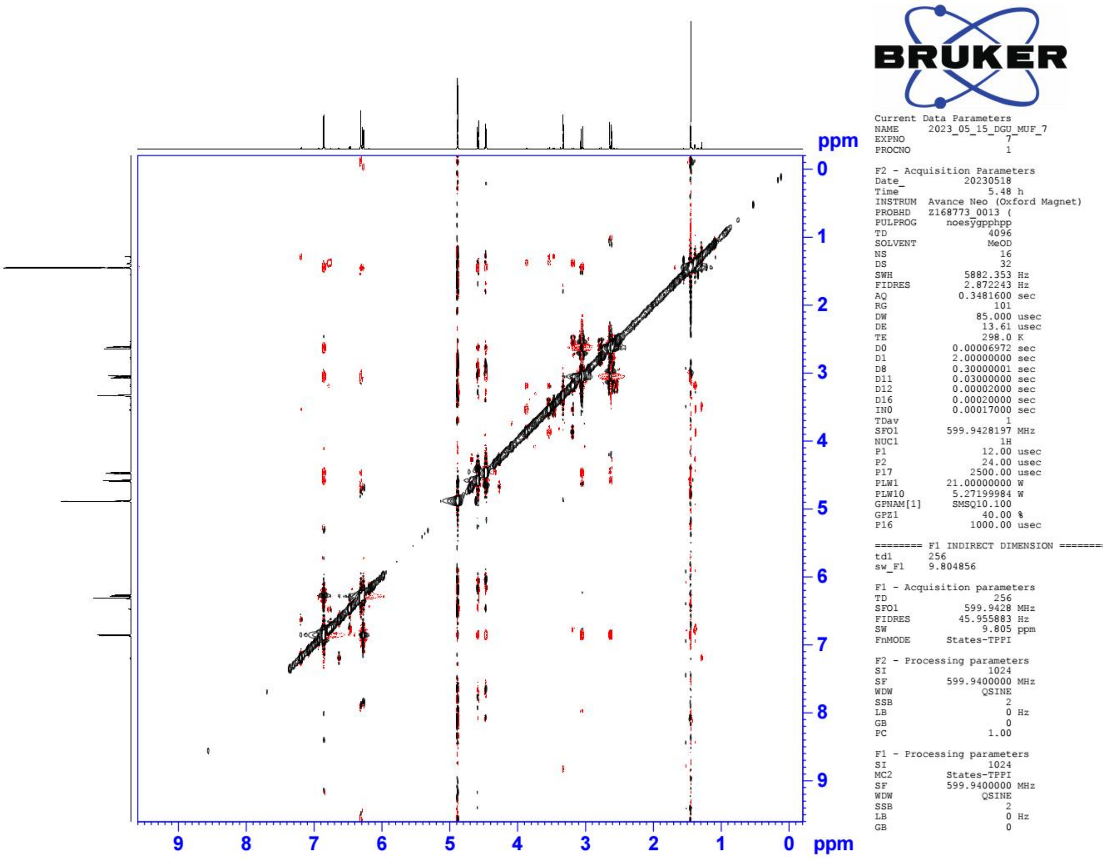

Figure S5. NOESY spectrum of compound **1** in CD_3OD.

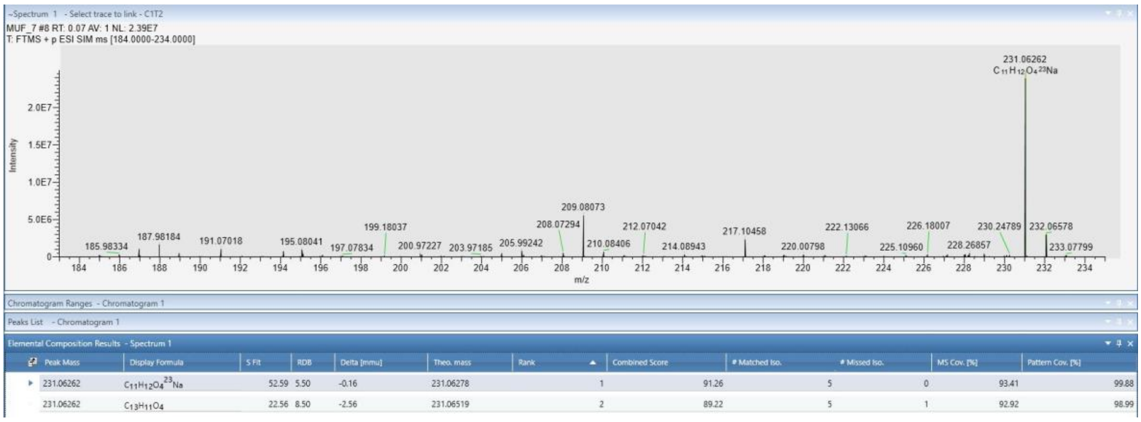

Figure S6. HRESIMS spectrum of compound **1**.

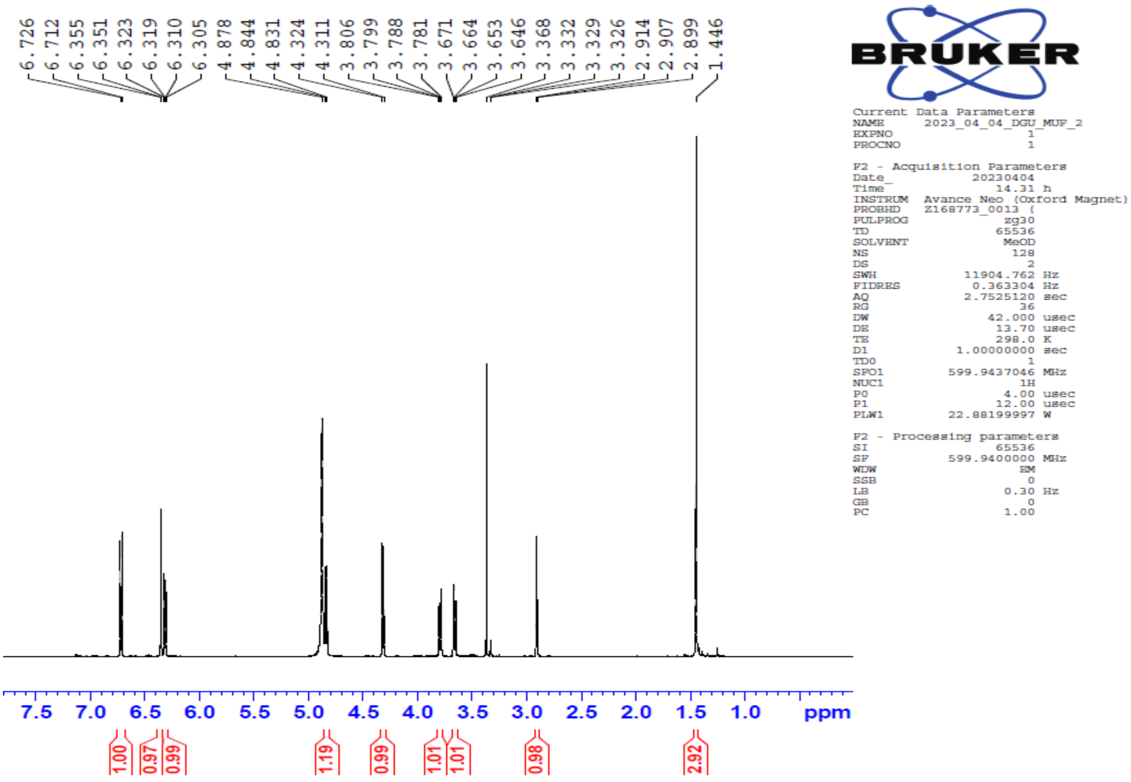

Figure S7. ^{1}H NMR spectrum of compound **2** in CD_3OD.

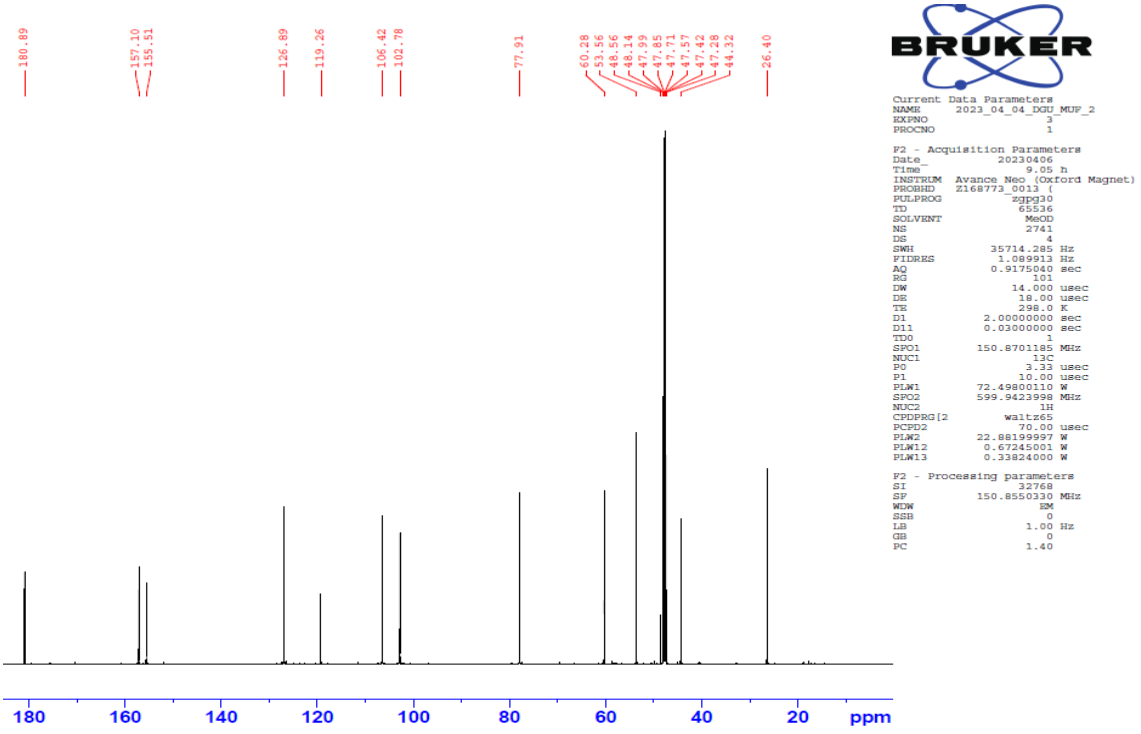

Figure S8. ^{13}C NMR spectrum of compound **2** in CD_3OD..

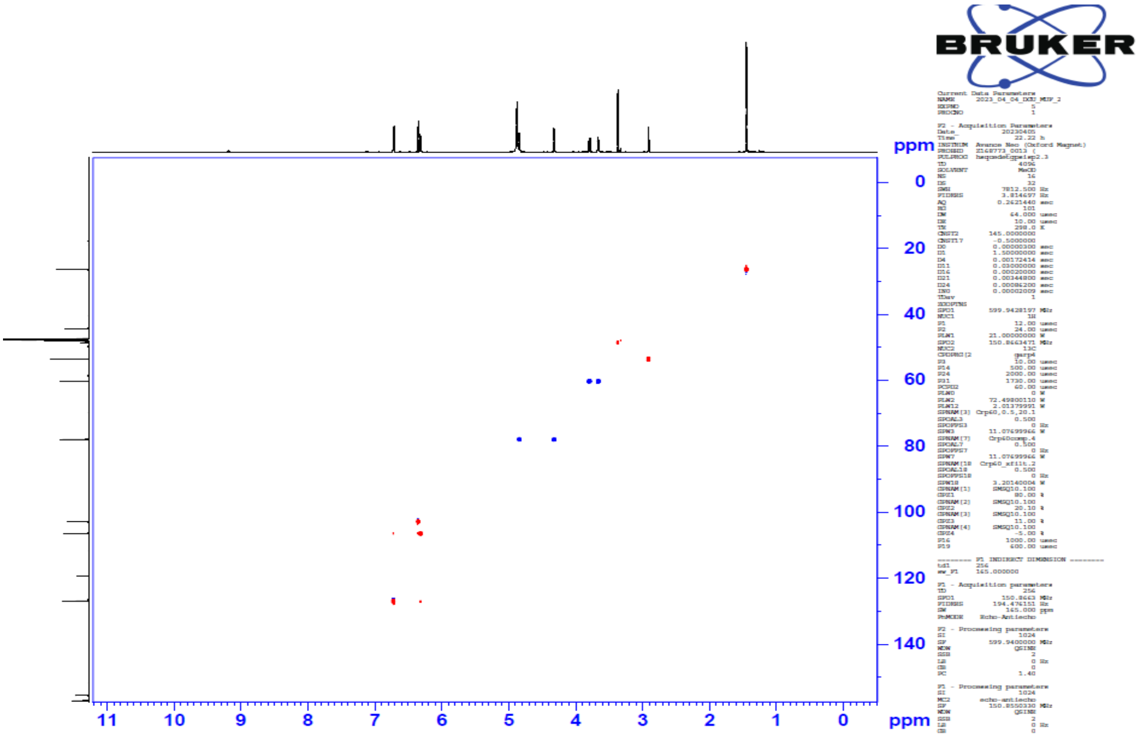

Figure S9. HSQC spectrum of compound **2** in CD_3OD.

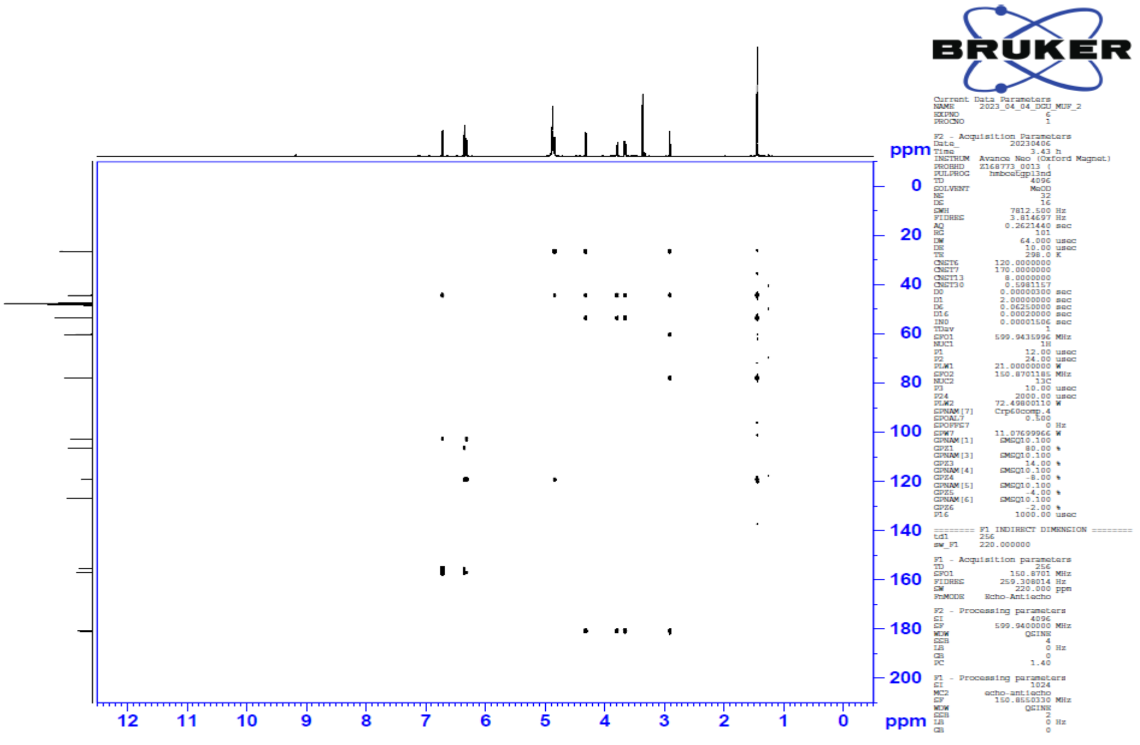

Figure S10. HMBC spectrum of compound **2** in CD_3OD.

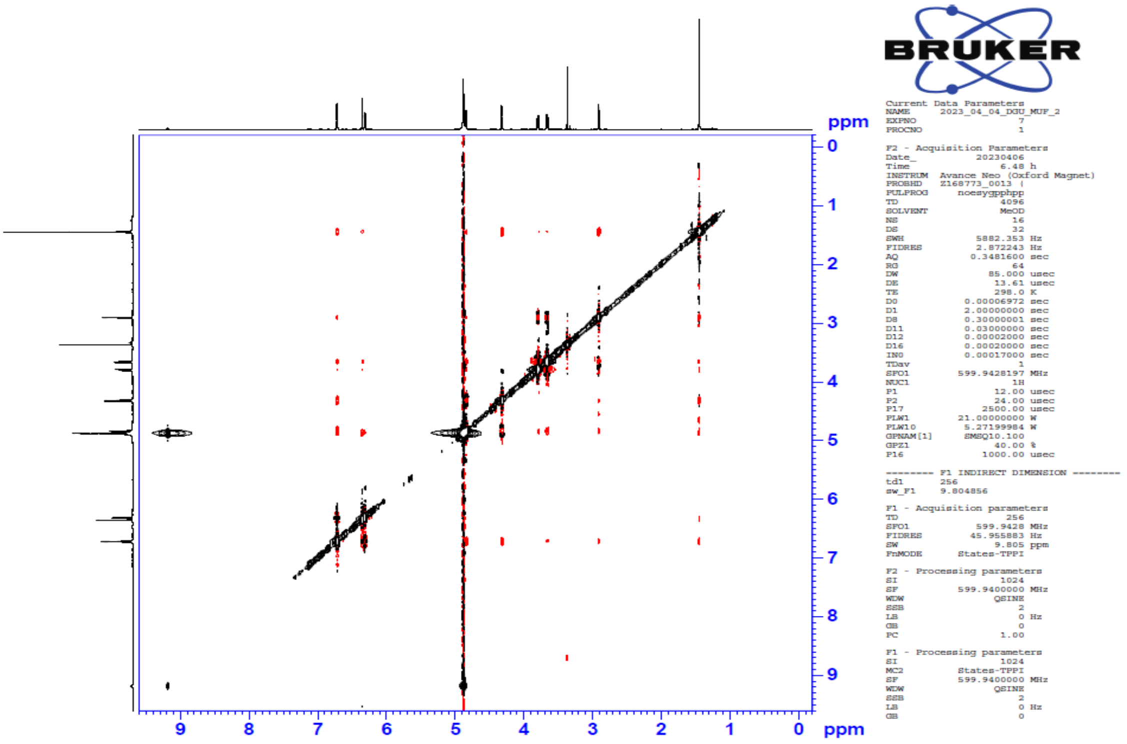

Figure S11. NOESY spectrum of compound **2** in CD_3OD.

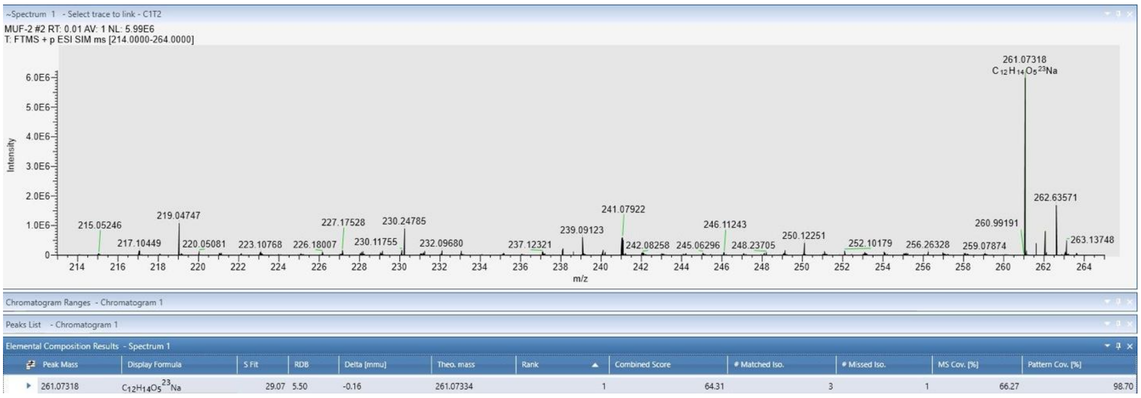

Peak Mass	Display Formula	S Fit	RDB	Delta [mmu]	Theo. mass	Rank	Combined Score	# Matched Iso.	# Missed Iso.	MS Cov. [%]	Pattern Cov. [%]
261.07318	$C_{12}H_{14}O_5{}^{23}Na$	29.07	5.50	-0.16	261.07334	1	64.31	3	1	66.27	98.70

Figure S12. HRESIMS spectrum of compound **2**.

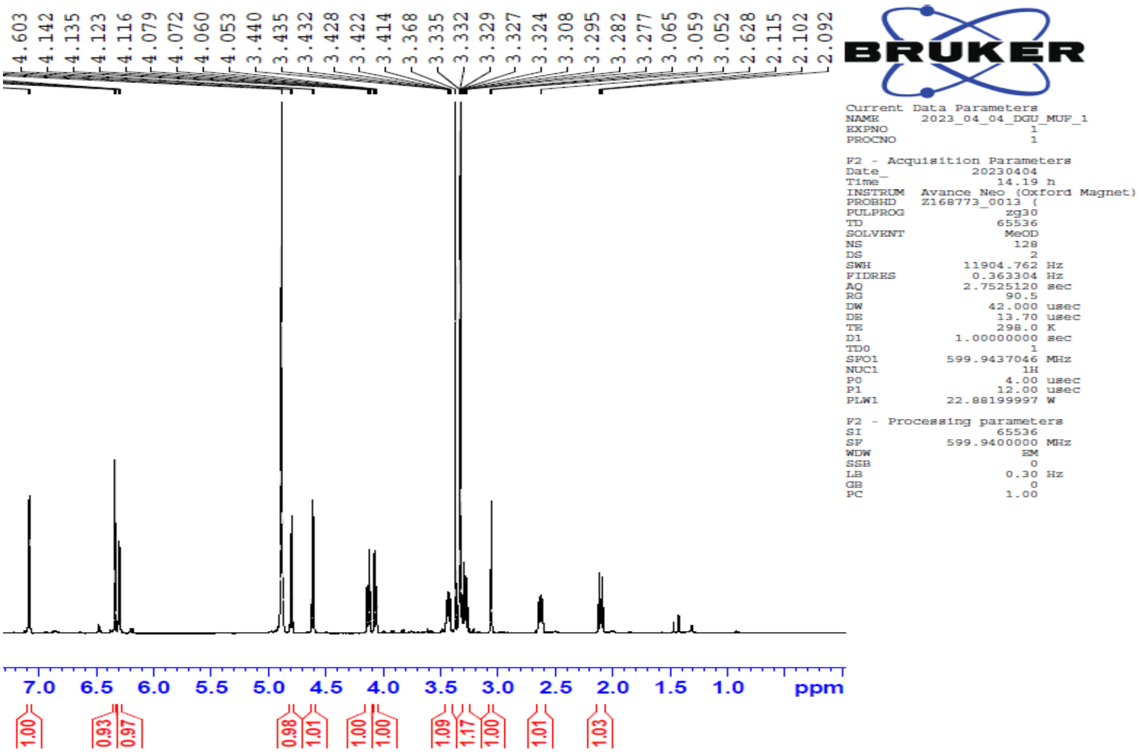

Figure S13. ^{1}H NMR spectrum of compound **3** in CD_3OD.

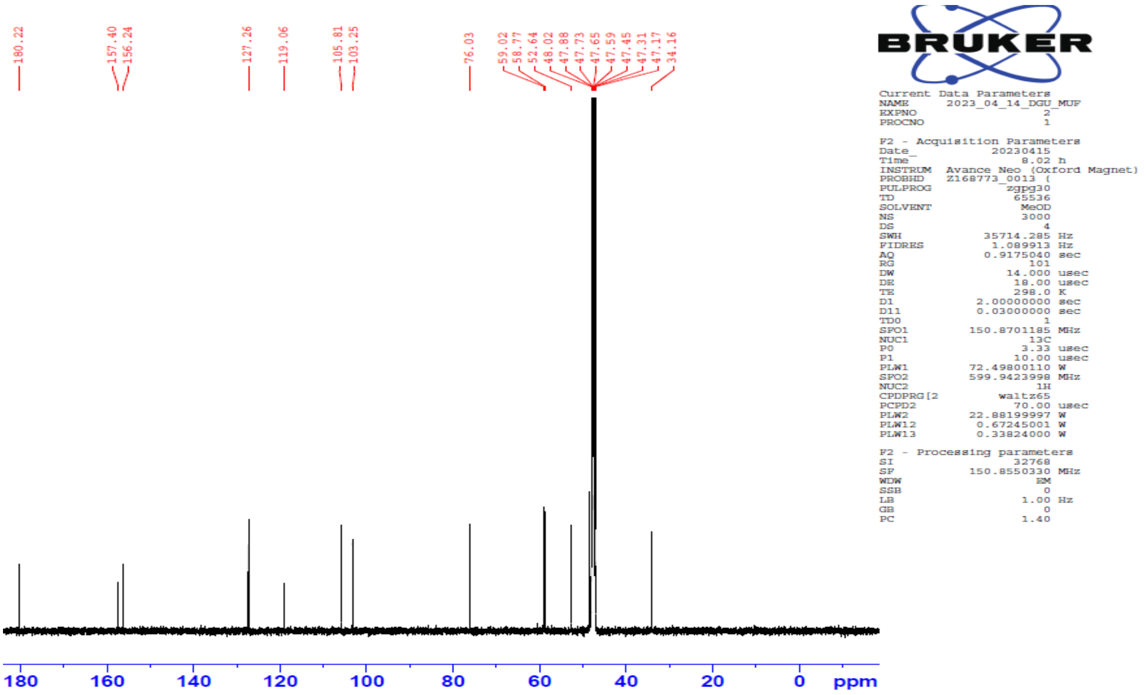

Figure S14. ^{13}C NMR spectrum of compound **3** in CD_3OD.

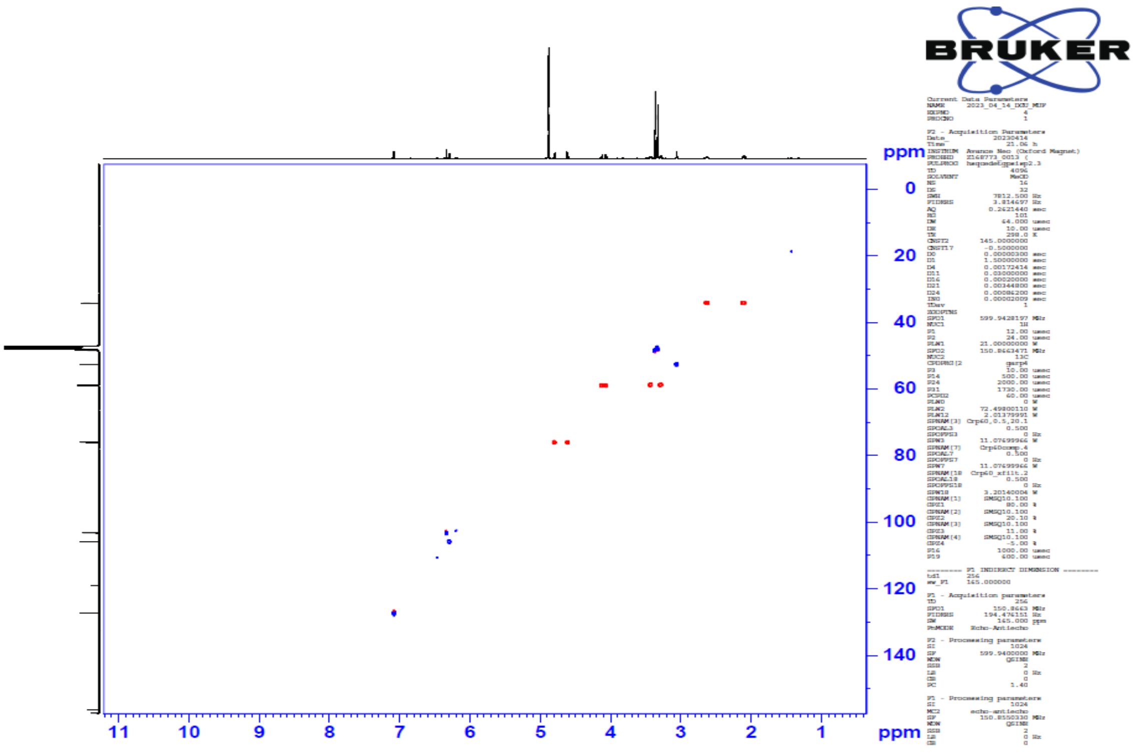

Figure S15. HSQC spectrum of compound **3** in CD_3OD.

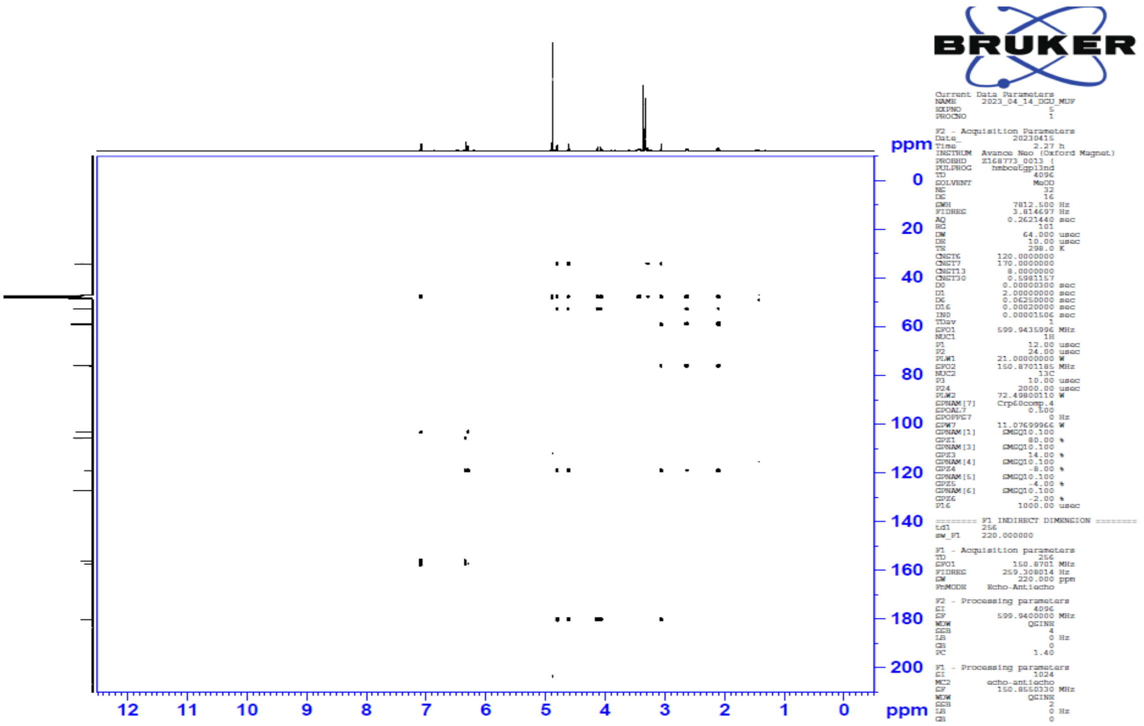

Figure S16. HMBC spectrum of compound **3** in CD_3OD.

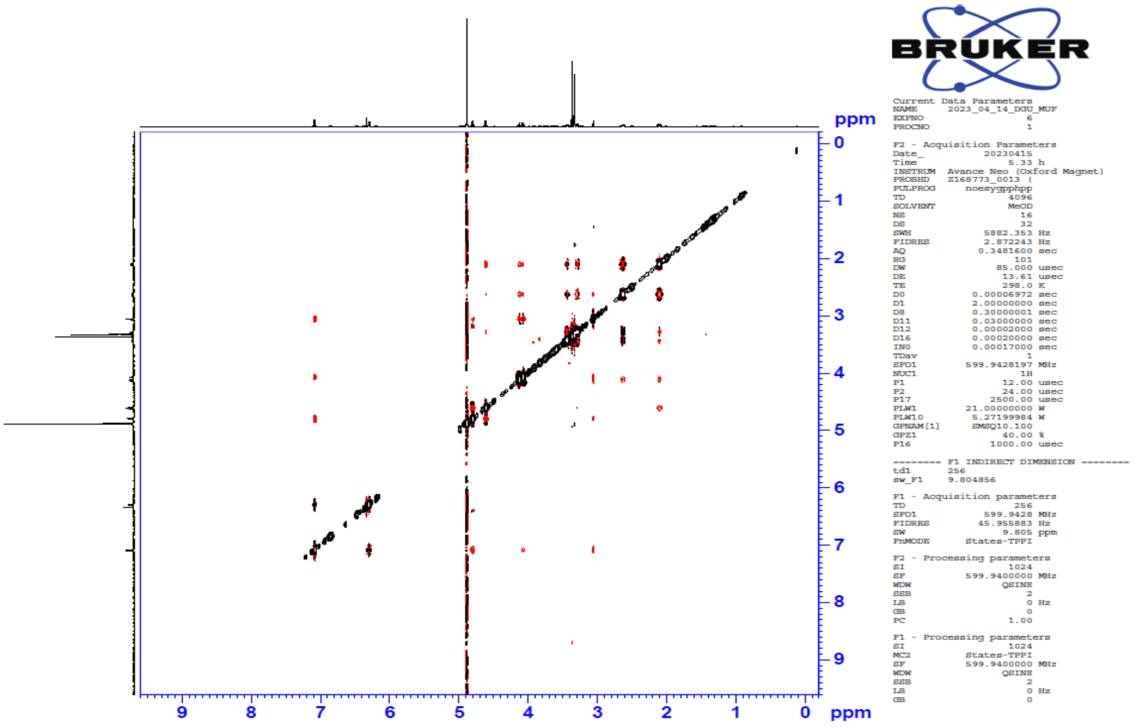

Figure S17. NOESY spectrum of compound **3** in CD_3OD.

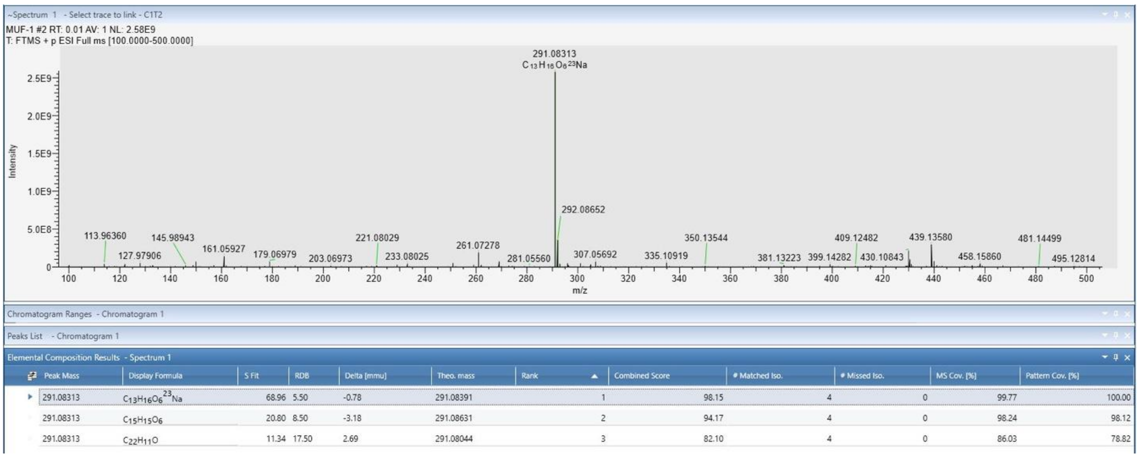

Figure S18. HRESIMS spectrum of compound **3**.

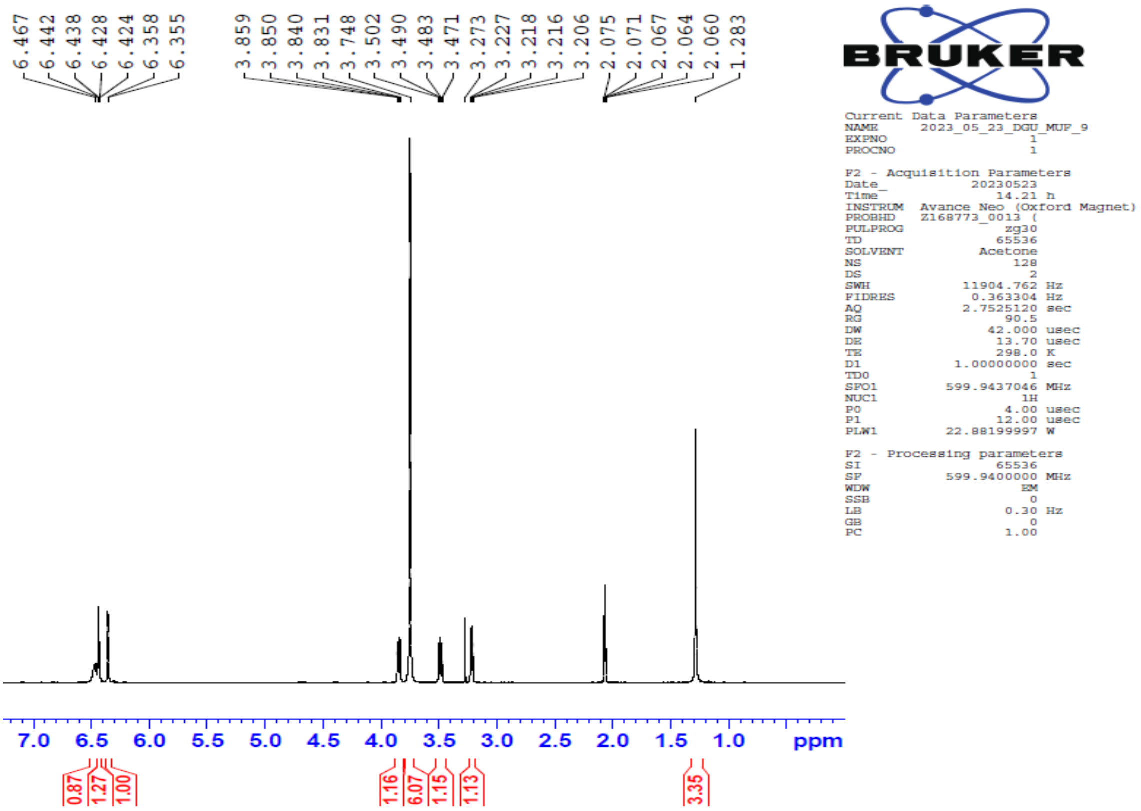

Figure S19. ^{1}H NMR spectrum of compound **4** in acetone-d_6+D_2O.

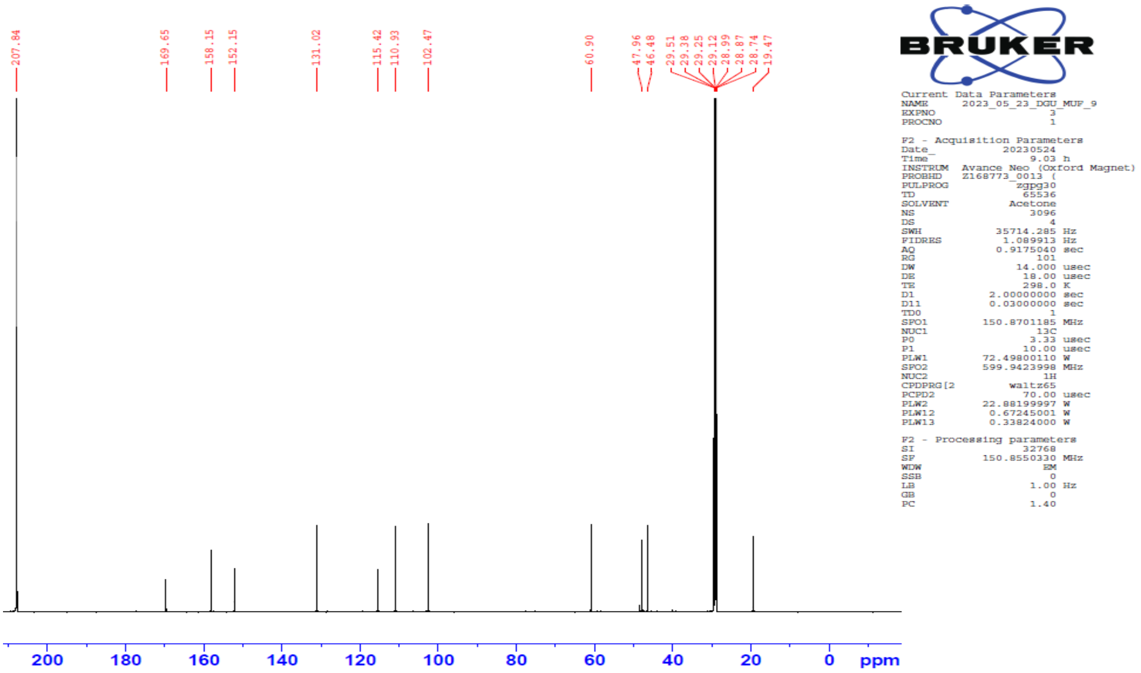

Figure S20. ^{13}C NMR spectrum of compound **4** in acetone-d_6+D_2O..

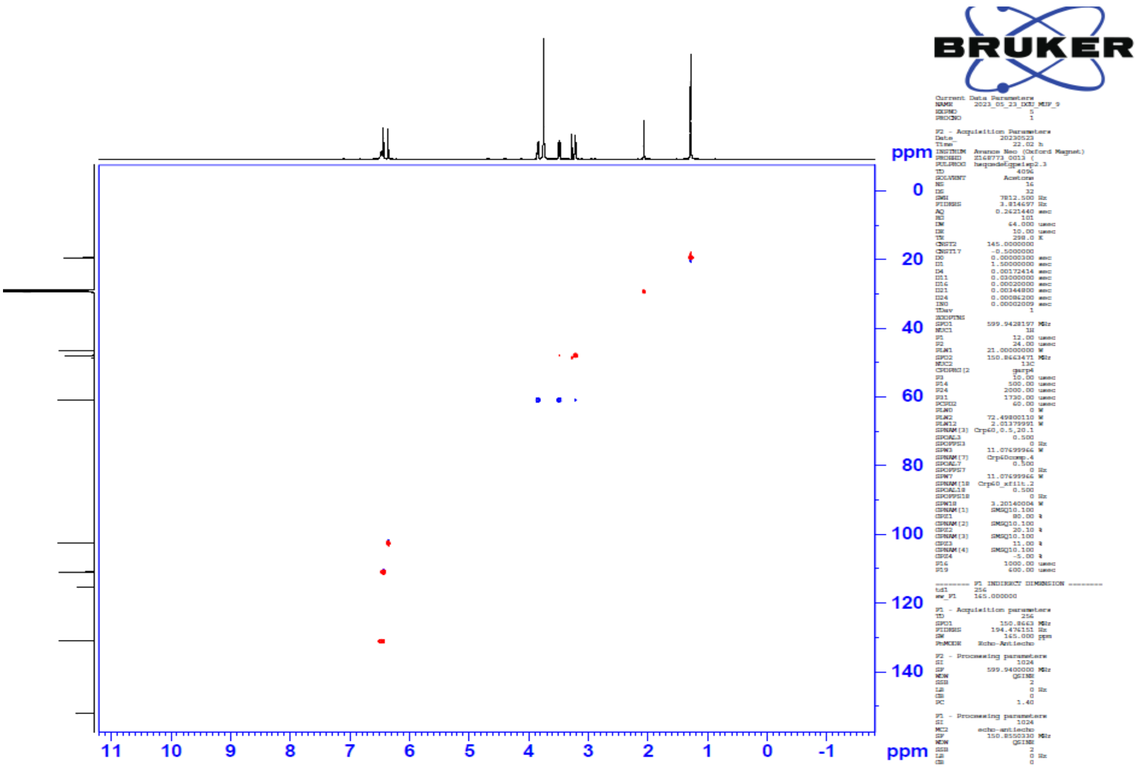

Figure S21. HSQC spectrum of compound **4** in acetone-d_6+D_2O..

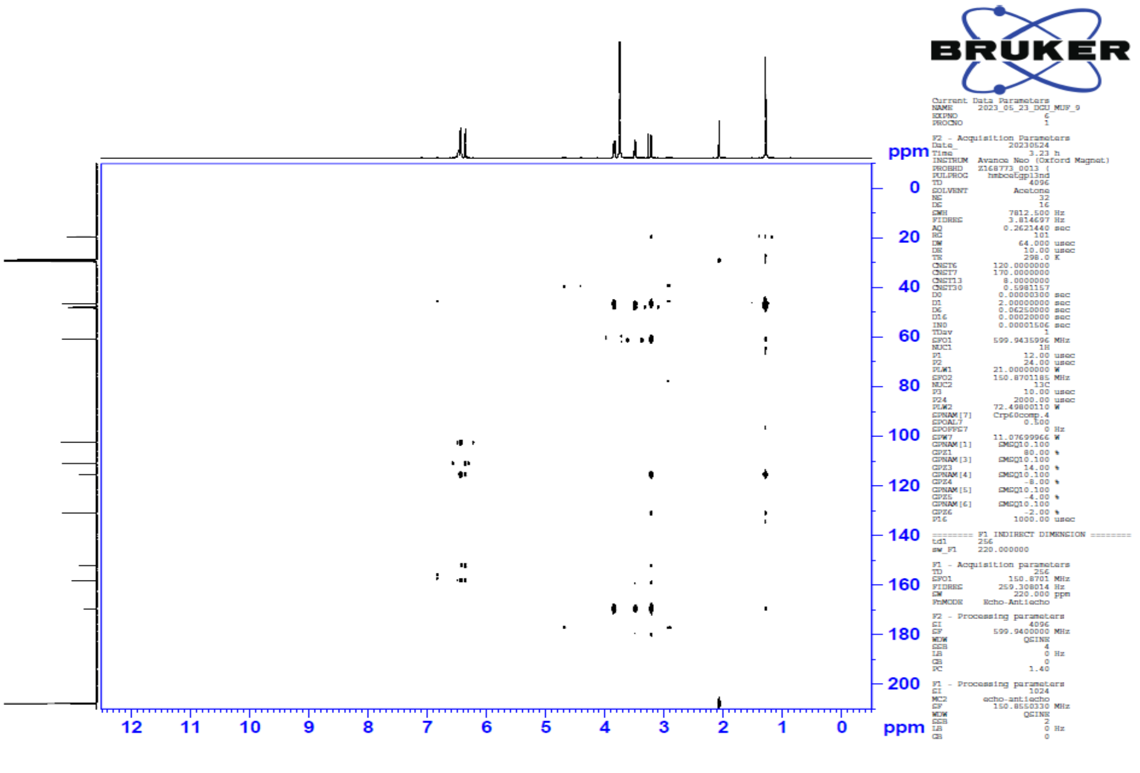

Figure S22. HMBC spectrum of compound **4** in acetone-d_6+D_2O.

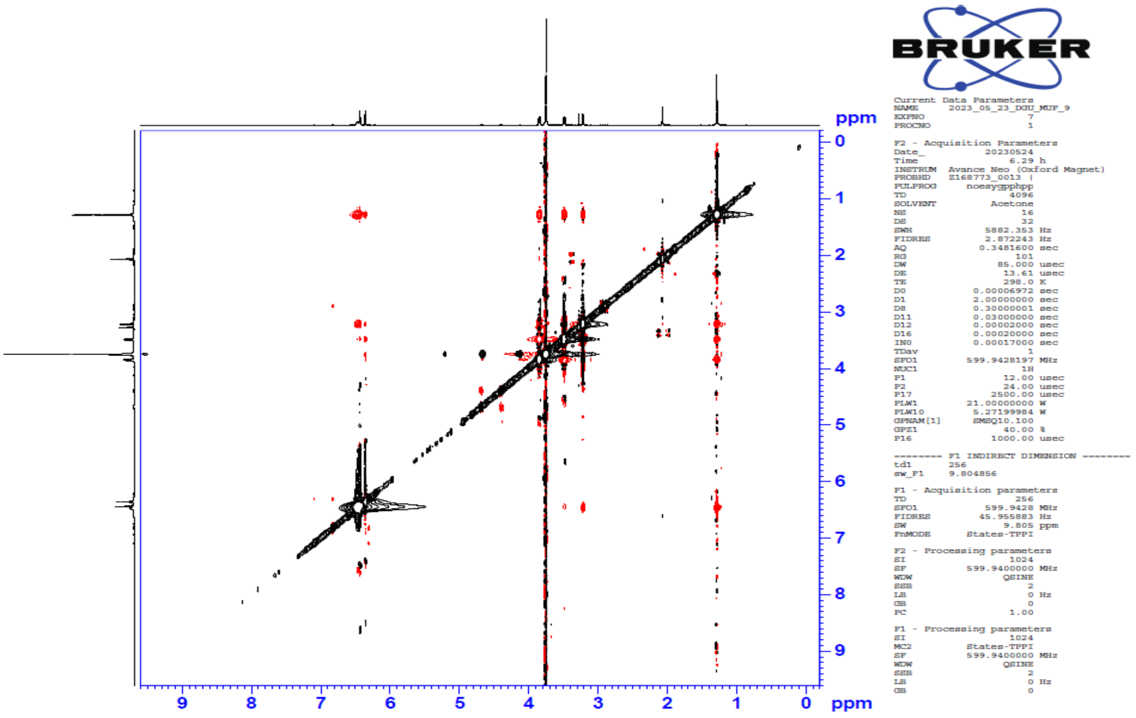

Figure S23. NOESY spectrum of compound **4** in acetone-d_6+D_2O.

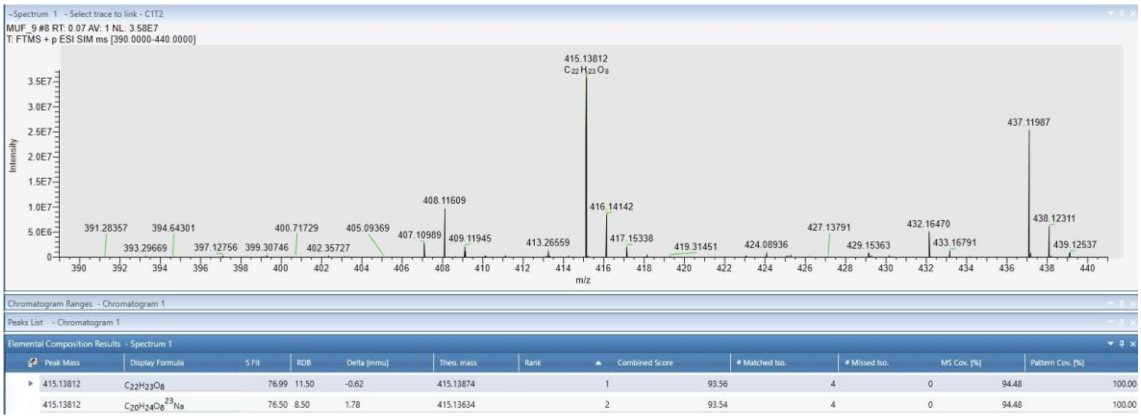

Peak Mass	Display Formula	S Fit	RDB	Delta [mmu]	Theo. mass	Rank	Combined Score	# Matched Iso.	# Missed Iso.	MS Cov. [%]	Pattern Cov. [%]
415.13812	$C_{22}H_{23}O_8$	76.99	11.50	-0.62	415.13874	1	93.56	4	0	94.48	100.00
415.13812	$C_{20}H_{24}O_8{}^{23}Na$	76.50	8.50	1.78	415.13634	2	93.54	4	0	94.48	100.00

Figure S24. HRESIMS spectrum of compound **4**.

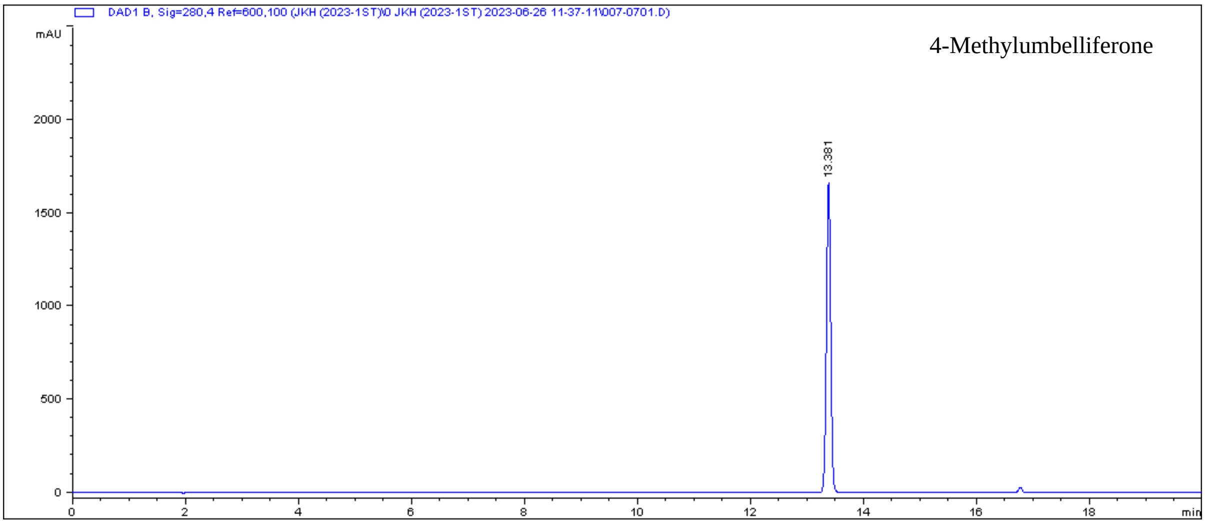
DAD1 B, Sig=280,4 Ref=600,100 (JKH (2023-1ST)\0 JKH (2023-1ST) 2023-06-26 11-37-11\007-0701.D)
4-Methylumbelliferone
mAU
13.381
min

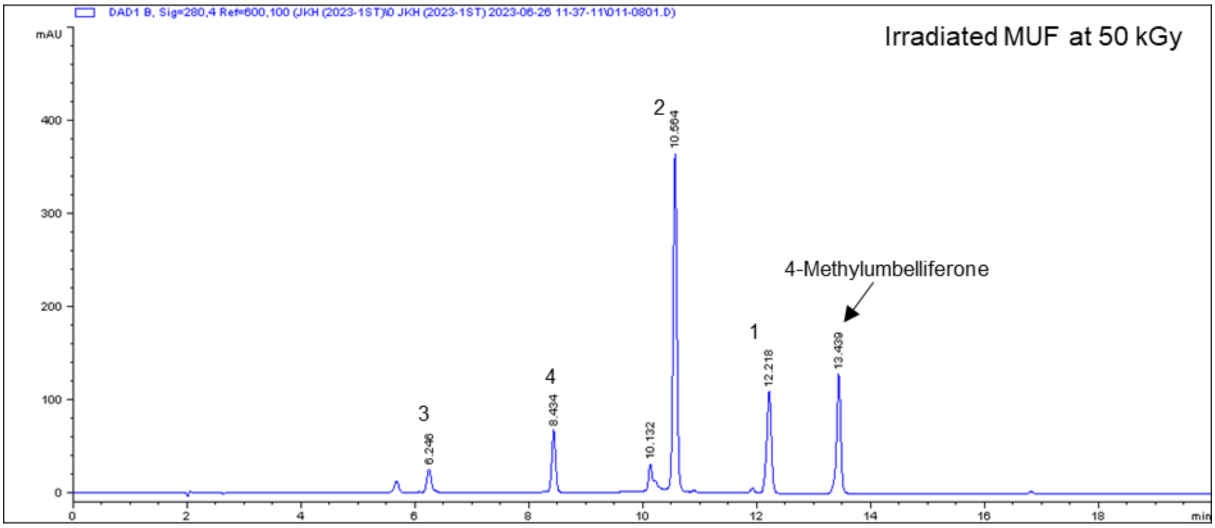
DAD1 B, Sig=280,4 Ref=600,100 (JKH (2023-1ST)\0 JKH (2023-1ST) 2023-06-26 11-37-11\011-0801.D)
Irradiated MUF at 50 kGy
mAU
4-Methylumbelliferone
1
2
3
4
6.246
8.434
10.132
10.564
12.218
13.439
min

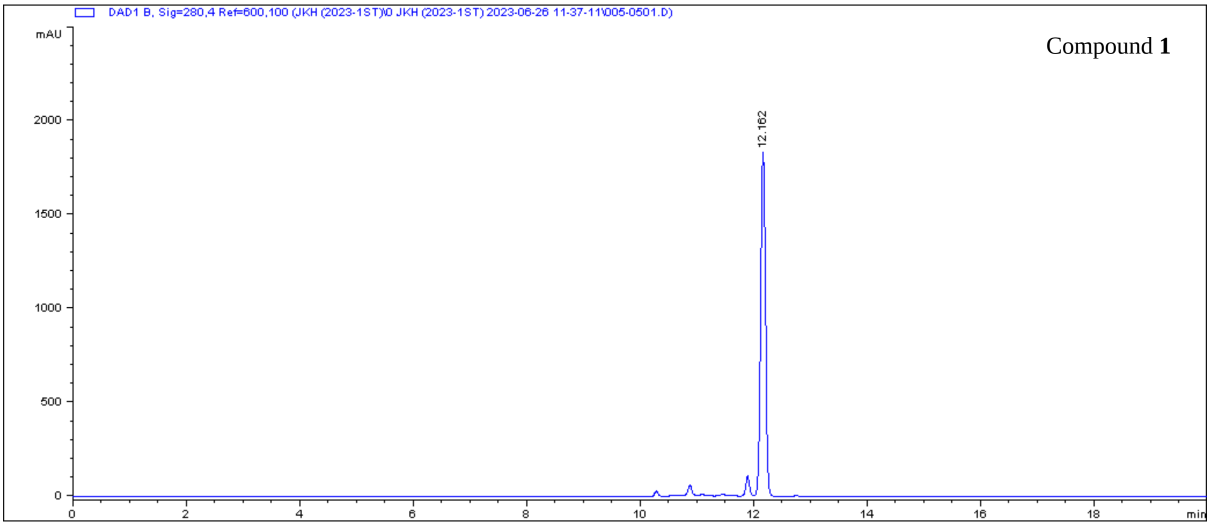
DAD1 B, Sig=280,4 Ref=600,100 (JKH (2023-1ST)\0 JKH (2023-1ST) 2023-06-26 11-37-11\005-0501.D)
Compound **1**
mAU
12.162
min

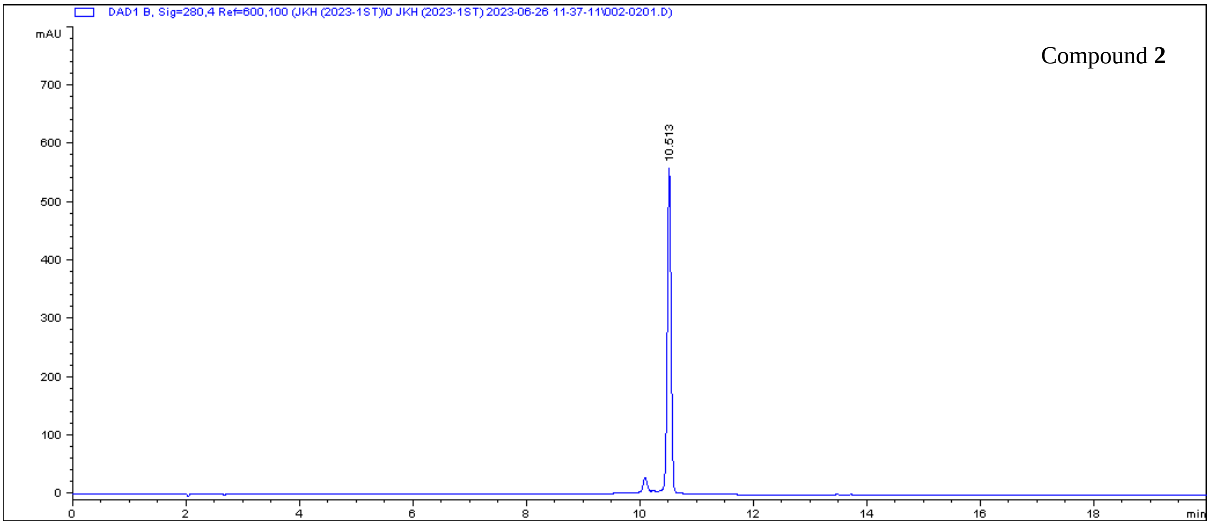

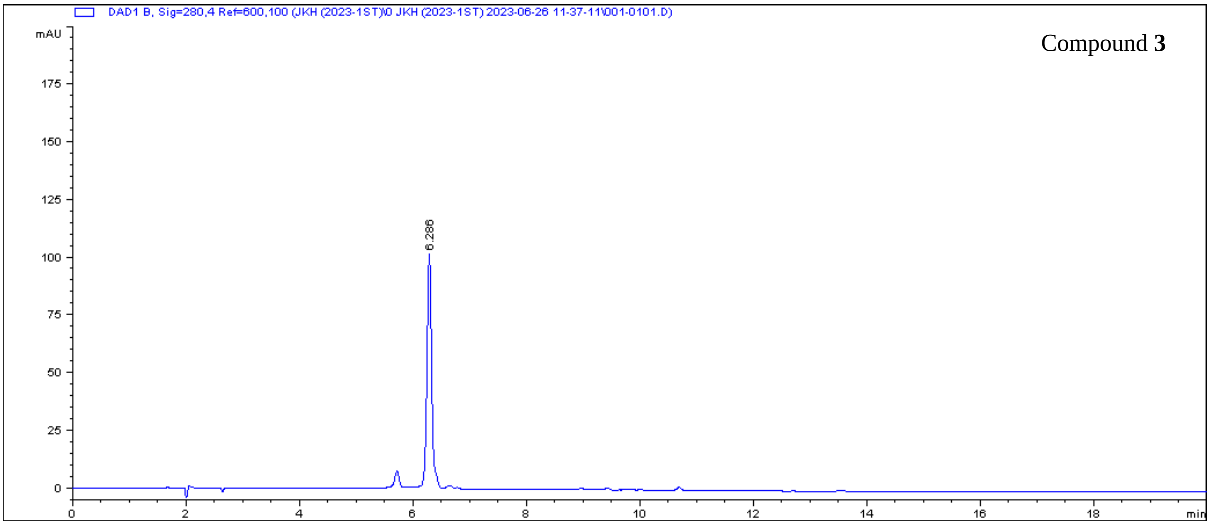

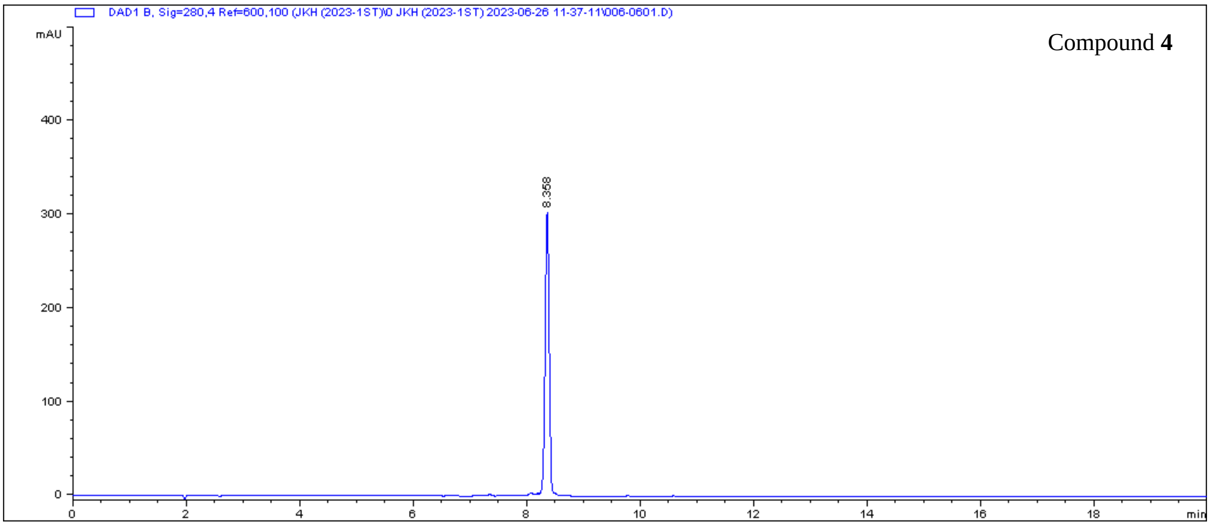

HPLC analysis was conducted on a YMC-Pack ODS A-302 column (4.6 mm i.d. × 150 mm; 5 μm particle size; YMC Co., Kyoto, Japan), and the gradient solvent system (detection: UV 280 nm; temperature: 40 ^{o}C) initiated with 0.1% HCOOH, increased to CH_3CN over 20 min and the flow rate was set at 1.0 mL/min.

Figure S25. HPLC chromatograms of gamma ray treated reactant and newly generated molecules **1–4**.

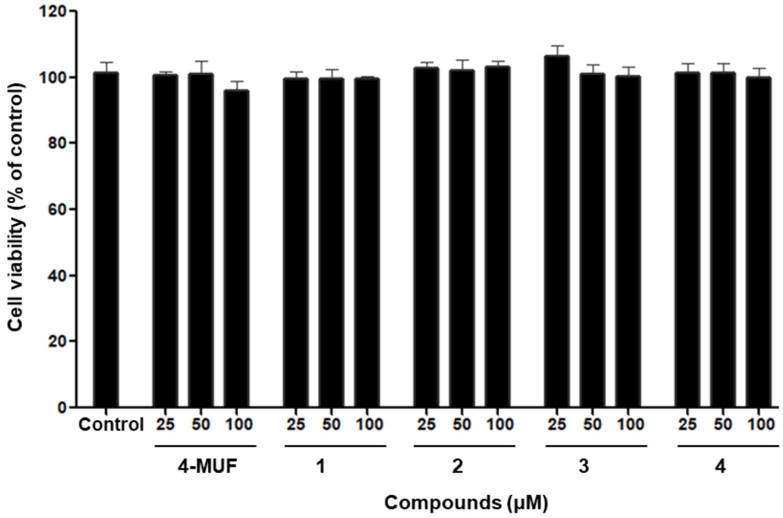

Figure S26. Effects of isolated compounds on the cell viability in B16F10 cells. Cell viability detection by MTT assay in B16F10 cells after treatment with extracts for 24 h.
